# Synergistic interaction between C5a and NOD2 signaling in the regulation of chemokine expression in RAW 264.7 macrophages

**DOI:** 10.4236/abb.2013.48A3004

**Published:** 2013-07-15

**Authors:** Hui Tang, Umme Amara, Dora Tang, Mark A. Barnes, Christine McDonald, Laura E. Nagy

**Affiliations:** 1 Departments of Pathobiology Cleveland Clinic, Cleveland, USA; 2 Department of Molecular Medicine, Case Western Reserve University, Cleveland, USA; 3 Department of Gastroenterology Cleveland Clinic, Cleveland, USA

**Keywords:** Anaphylatoxin, C5L2, C5a Receptor, Complement, NOD2

## Abstract

The innate immune response is a complex process involving multiple pathogen-recognition receptors, including toll-like receptors (TLRs) and nucleotide-binding oligomerization domain (NOD)-like receptors. Complement is also a critical component of innate immunity. While complement is known to interact with TLR-mediated signals, the interactions between NOD-like receptors and complement are not well understood. Here we report a synergistic interaction between C5a and Nod2 signaling in RAW 264.7 macrophages. Long-term treatment with muramyl dipeptide (MDP), a NOD2 ligand, enhanced C5a-mediated expression of chemokine mRNAs in RAW 264.7 cells. This response was dependent on NOD2 expression and was associated with a decrease in expression of C5L2, a receptor for C5a which acts as a negative modulator of C5a receptor (C5aR) activity. MDP amplified C5a-mediated phosphorylation of p38 MAPK. Treatment of RAW264.7 cells with an inhibitor of p38 attenuated the synergistic effects of C5a on MDP-primed cells on MIP-2, but not MCP-1, mRNA. In contrast, inhibition of AKT prevented C5a stimulation of MCP-1, but not MIP-2, mRNA, in MDP-primed cells. Taken together, these data demonstrated a synergistic interaction between C5a and NOD2 in the regulation of chemokine expression in macrophages, associated with a down-regulation of C5L2, a negative regulator of C5a receptor activity.

## 1. INTRODUCTION

The host response to pathogen infection and/or endogenous danger signals is a complex process in which multiple components of the innate immune response are activated to precisely control innate immunity. Cell-based pathways, including pathogen-recognition receptors (PRRs), as well as circulating innate immune pathways, such as complement, are activated by pathogens/danger signals, inducing inflammatory responses in response to infection and/or tissue injury. While individual signaling pathways for PRRs and complement are relatively well understood, the complex interactions between these different arms of the innate immune response have been less well studied.

Proteolysis of C5 is one of the key events of complement activation, generating small cleavage fragment, C5a. C5a, an anaphylatoxin, induces or amplifies multiple innate immune responses. C5a exerts most of its functions through interaction with its cognate receptor, C5a receptor (C5aR). However, an emerging body of evidence indicates the involvement of a second C5a receptor, C5L2, which acts as a negative modulator of C5aR activity [1]. C5aR and C5L2 share significant homology and belong to G-protein coupled receptor (GPCR) family [2]. In coordination with C5aR, C5L2 is expressed on macrophages, dendritic cells and neutrophils [3]. C5a binding to C5aR induces rapid auto-phosphorylation, internalization and subsequent signaling leading to acute inflammatory responses [4]. Accumulating evidences suggest that C5L2 is a functional receptor for C5a. However, depending on the specific cellular/ tissue environment, C5L2 may be either anti-inflammatory or pro-inflammatory [4].

Anaphylatoxin signaling interacts with signaling via the toll-like receptors (TLRs). For example, when cells are exposed to both C5a and LPS, the prototypical TLR4 ligand, production of numerous of cytokines and chemokines is synergistically enhanced [5]. This synergism between C5aR and TLR4 is negatively modulated by C5L2 [1].

Interactions between anaphylatoxin signaling and nucleotide-binding oligomerization domain (NOD)-like receptors, another major class of pattern-recognition receptors [6], have not been investigated. The NOD proteins, NOD1 and NOD2, are primarily expressed by antigen-presenting cells and epithelial cells, functioning as cytosolic sensors for innate recognition of microorganisms; recognition results in the up-regulation of inflammatory responses, as well as induction of apoptosis [7]. Importantly, Nod2 is involved in the pathogenesis of Crohn’s disease [8,9]. NOD2 consists of a C-terminal leucine-rich repeat (LRR) domain, a central NOD domain and an N-terminal caspase-recruitment domain (CARD) [10]. The primary NOD2 ligand is muramyl dipeptide (MDP), a component from both Gram-positive and Gram-negative bacteria [11]. MDP recognizes LRR domains, leading to Nod2 activation, which then recruits receptor-interacting serine/threonine kinase (RICK/RIP2) via CARD-CARD interactions. Activation of RIP2 switches on nuclear factor-κB (NF-κB), a transcription factor, and the mitogen-activated protein kinases (MAPKs), including p38 MAPK, c-Jun N-terminal kinase (JNK) and extracellular signal regulated kinases 1 and 2 (ERK1/2). The net response to this signaling is a potent induction of pro-inflammatory cytokine expression [10, 12,13].

Here we tested the hypothesis that NOD2 and C5a-signaling pathways will interact in the regulation of chemokine expression in macrophages. Indeed, there was a positive modulatory effect of NOD2 activation on the sensitivity of macrophages to C5a. Further, MDP priming of macrophages was associated with a suppression in the expression of C5L2. These data thus characterize a novel mechanism for cross-talk between NOD2 and C5a-mediated signaling and identify the regulation of C5L2 expression as a potential site for therapeutic interventions in the treatment and/or prevention of inflammatory conditions.

## 2. MATERIALS AND METHODS

### 2.1. Materials

The murine RAW 264.7 cell line was purchased from the American Type Culture Collection (ATCC, Rockville, MD). Cell culture reagents were obtained from GIBCO (Grand Island, NY). Antibodies were purchased from the following sources: C5aR (Santa Cruz Biotechnology, Santa Cruz, CA), C5L2 (R & D Systems, Inc., Minneapolis, MN), Syntaxin-6 (BD Transduction Laboratories, San Diego, CA), heat shock protein 70 (HSC70) (Santa Cruz Biotechnology), phospho-AKT (cell signaling), phospho-p38 (Santa Cruz Biotechnology), phospho-ERK1/2 (cell signaling) and total ERK1/2 (Upstate Biotechnology; Lake Placid, NY). Alexa fluor-488-conjugated and Alexa fluor-568-conjugated secondary antibody were purchased from Invitrogen (Carlsbad, CA). Protease inhibitor cocktail was purchased from *Boehringer Mannheim* (Indianapolis, IN). LPS from *E. coli* serotype 026:B6 (tissue culture-tested, L-2654) was purchased from Sigma. All experiments were carried out with a single lot of LPS (lot number 064K4077). C5a was purchased from R&D Systems, Inc. MDP was purchased from Bachem (Torrance, CA). Validated Silencer Select siRNA pre-designed sequences were purchased from Ambion/Applied Biosystems. General research *chemicals* were purchased from Sigma-Aldrich (St. Louis, MO).

### 2.2. Culture and Nucleofection of RAW 264.7 Cell Macrophages

The murine RAW 264.7 macrophage-like cell line was routinely cultured in DMEM with 10% fetal bovine serum and penicillin/streptomycin at 37°C and 5% CO_2_. RAW 264.7 cells were transfected using the Amaxa nucleofector apparatus (Lonza, Cologne, Germany). Briefly, 2 × 10^6^ cells were resuspended in 100 µl of nucleofector solution and were nucleofected with 200 nM of specific or scrambled siRNA, or 2 µg DNA in the nucleofector device using the D-032 program, according to the instructions of the manufacturer. Transfected cells were seeded at 5.0 × 10^5^/well in 96-well plates and cultured for 32 h prior to experimental treatments. Efficiency of knockdown was determined by quantitative real time polymerase chain reaction (RT-PCR).

### 2.3. Western Blotting and Protein Concentration Assay

Protein concentrations were measured using a BCA kit (Pierce, Rockford, IL). Western-blot was performed using enhanced chemiluminescence. Signal intensities were quantified by densitometry using image J software (NIH).

### 2.4. RT-PCR

Total RNA was isolated from murine RAW 264.7 macrophage-like cell line using the RNeasy mini kit (Qiagen, Valencia, CA), following the manufacturer’s instructions. Total RNA was reverse transcribed using a RETROscript kit (Ambion, Austin, TX). Quantitative RT-PCR amplification was performed using the Brilliant SYBR green QPCR Master Mix (Applied Biosystems, Warrington, UK) in an Mx3000p PCR machine (Stratagene, La Jolla, CA) as previously described [14]. The primer sequences used were:

**RT-PCR primers**

**Table:** 

Genes	Forward primer	Reverse primer
18S	ACGGAAGGGCACCACCAGGA	CACCACCACCCACGGAATCG
MCP-1	AGGTCCCTGTCATGCTTCTG	TCTGGACCCATTCCTTCTTG
MIP-2	GCGCCCAGACAGAAGTCATAG	AGCCTTGCCTTTGTTCAGTATC
TNFα	CCCTCACACTCAGATCATCTTCT	GCTACGACGTGGGCTACAG
Nod2	CGACATCTCCCACAGAGTTGTAATCC	GGCACCTGAAGTTGACATTTTGC
C5L2	ACCACCAGCGAGTATTATGACT	GCTGCATACAGCACAAGCA
C5aR	GTGGGTTTTGTGTTGCCTCT	TGATAGGGCAGCCAGAAGAT

### 2.5. Immunohistochemistry

RAW 264.7 cells were plated on a chamber slide (Nunc, Rochester, NY) with cell density of 0.1 × 10^6^ cells/mL, 0.5 mL/well. Cells were stimulated as indicated in figure legends and then washed with ice-cold phosphate buffered saline (PBS). Cells stained with or without CM-Dil (Invitrogen, Eugene, OR), a cell membrane marker. Cell were fixed with 4% paraformaldehyde for 20 min at room temperature and quenched with 25 mM glycine. Cells were blocked with 1% fish gelatin containing 2% bovine serum albumin (BSA) with 0.1% Triton-X-100 for 1 h and incubated overnight with anti-C5aR (1:50) or anti-C5L2 (1:50),and anti-syntaxin-6 (1:20), at 4°C in a humidified chamber. After 3 washes in PBS buffer, cells were incubated with fluorochrome-conjugated secondary antibody for 1 h at room temperature. Cells were washed 3 times in PBS buffer and mounted with VECTAS-HIELD™. A slide without primary antibody was used as negative control. Fluorescence images were acquired using a LEICA confocal microscope (Leica Microsystems, Inc., Buffalo Grove, IL).

### 2.6. Flow Cytometry Analysis

After 16 h of culture with or without MDP, RAW264.7 cells were gently scraped and adjusted to 1 × 10^6^ cells/ml with culturing media. The cells were centrifuged at 100×g for 10 min. The pellet was washed with PBS and re-suspended in PBS with 0.1% sodium azide and then blocked with 0.5 µg of anti-mouse CD32/CD16 Fcγ receptor blocking antibody (eBioscience, San Diego, CA) for 15 min at room temperature. Cells were then stained with 0.5 µg of phycoerythrin-conjugated (PE) C5L2 (Biolegend, San Diego, CA), C5aR (AbD Serotec, Oxford, UK), or isotype control, PE-conjugated IgG2a diluted in PBS containing 0.1% sodium azide for 30 min. Cells were washed twice with PBS, resuspended in 0.5 ml of wash buffer (final concentration 1 × 10^6^ cells in 0.5 ml), and fixed overnight at 4°C until flow cytometric measurements were performed on a LSRII flow cytometer (BD Biosciences, San Jose, CA). Data were acquired and processed using FlowJo software (Tree Star, Inc., Ashland, OR).

### 2.7. Statistical Analysis

Values reported are means ± standard error of mean. Data were analyzed by Student’s t-test or ANOVA (general linear models procedure) (SAS Institute, Cary, IN) followed by least square means between groups. *P* values of less than 0.05 were considered significant.

## 3. RESULTS

**Figure 1:**
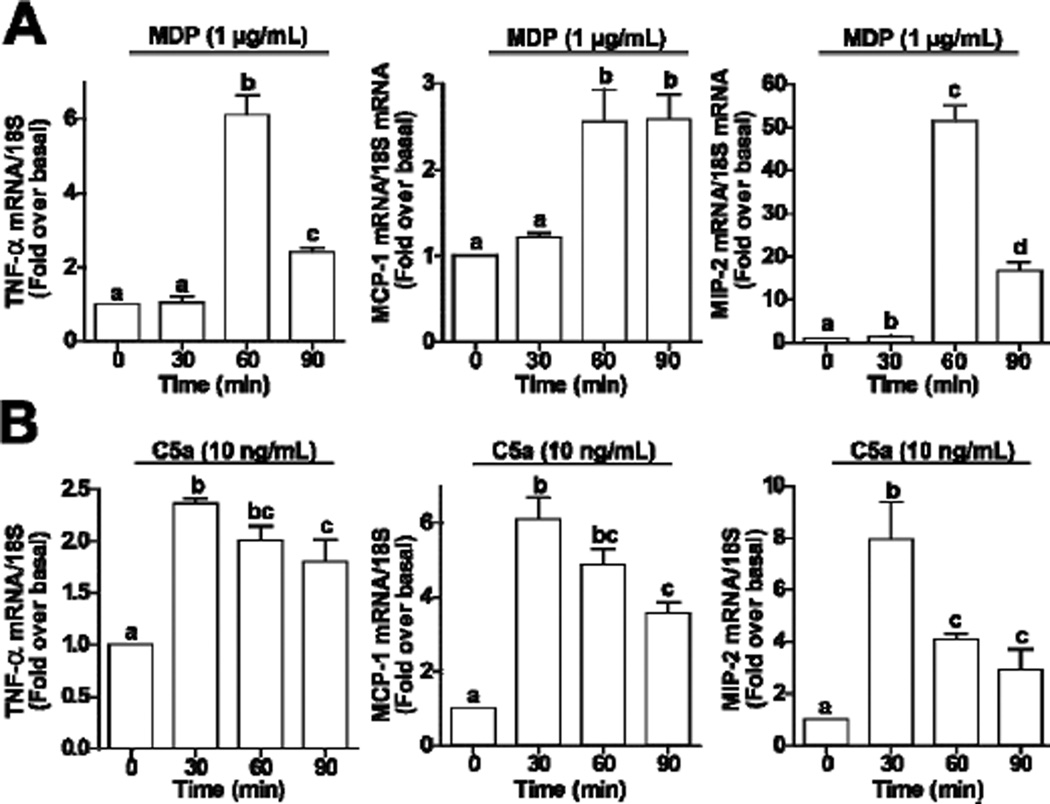
MDP and C5a increase expression of TNF-α, MCP-1 and MIP-2 mRNA in RAW 264.7 macrophages. RAW 264.7 macrophages were challenged with (A) MDP (1 µg/mL) or (B) C5a (10 ng/mL) for 0 – 90 min. Expression of TNF-α, MCP-1 and MIP-2 mRNA was measured by qRT-PCR. Values represent means ± SEM, *n* = 4. Values with different superscripts are significantly different from each other (*p* < 0.05).

Challenge of RAW 264.7 macrophages with C5a or MDP increased expression of cytokine and chemokine mRNA. Treatment with 1 µg/ml MDP, a ligand for NOD2, increased expression of TNFα mRNA after 60 min (Figure 1A). Similarly, induction of both MCP-1 and MIP-2 were increased by 60 min after stimulation with MDP. The stimulation was transient for TNFα and MIP-2, decreasing by 90 min, while MCP-1 expression remained elevated even after 90 min (Figure 1A). The response to challenge with C5a was more rapid, with peak TNFα, MCP-1 and MIP-2 expression observed by 30 min; expression then declined over 90 min (Figure 1B).

**Figure 2:**
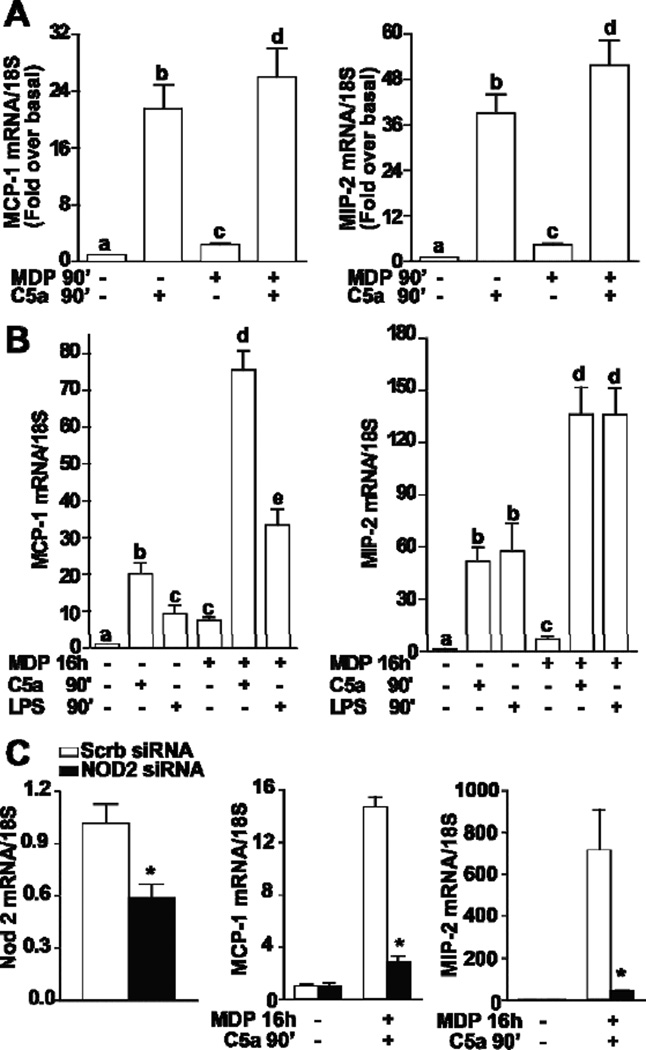
MDP-mediated sensitization of RAW 264.7 macrophages to subsequent stimulation with C5a is dependent on NOD2 expression. RAW 264.7 macrophages were treated with or without MDP (1 µg/mL) for (A) 90 min or (B) 16 h. Cells were then challenged with C5a (10 ng/mL) for 90 min and expression of MCP-1 and MIP-2 mRNA measured by qRT-PCR. (C) RAW 264.7 macrophages were nucleofected with 200 nM of Nod2 siRNA or scrambled siRNA. The efficiency of siRNA was assessed by mesauring NOD2 mRNA. After 32 h, cells were then treated with or without MDP (1 µg/mL) for an additional 16 h. Cells were then challenged with C5a (10 ng/mL) for 90 min and expression of MCP-1 and MIP-2 mRNA measured by qRT-PCR. Values represent means ± SEM, *n* = 3–5. (A/B) Values with different superscripts are significantly different from each other, *p* < 0.05. (C) *p < 0.05 compared to cells transfected with scrambled siRNA.

In order to evaluate a potential interaction between NOD2 signaling and C5a-mediated pro-inflammatory responses, cells were challenged with MDP and C5a simultaneously for 90 min. While both MDP and C5a alone increased MCP-1 and MIP2 mRNA expression at 90 min, there was only a minimal synergy between the two pathways when both agents were added together (Figure 2A). In contrast, when RAW 264.7 macrophages were pre-treated with MDP for 16 h, their sensitivity to subsequent challenge with C5a was increased, resulting in enhanced C5a-stimulated expression of MCP-1 and MIP-2 mRNA (Figure 2B).

In order to determine if NOD2 signaling was required for this interaction between MDP- and C5a-mediated chemokine expression, Nod2 was knocked down by transfecting Nod2 siRNA into the cell. Nod2 mRNA expression was reduced by 60% in cells transfected with Nod2 siRNA compared to cells transfected with scrambled siRNA (Figure 2C). Knocking down Nod2 in the cell prevented the synergism between MDP- and C5a-induced expression of chemokine mRNA (Figure 2C); the synergistic effect was sustained in cells transfected with scrambled siRNA. These data demonstrate that, in addition to the known synergism between TLR4 and NOD2 signaling [15], there is also a synergistic interaction between C5a and NOD2 signaling in macrophages.

**Figure 3:**
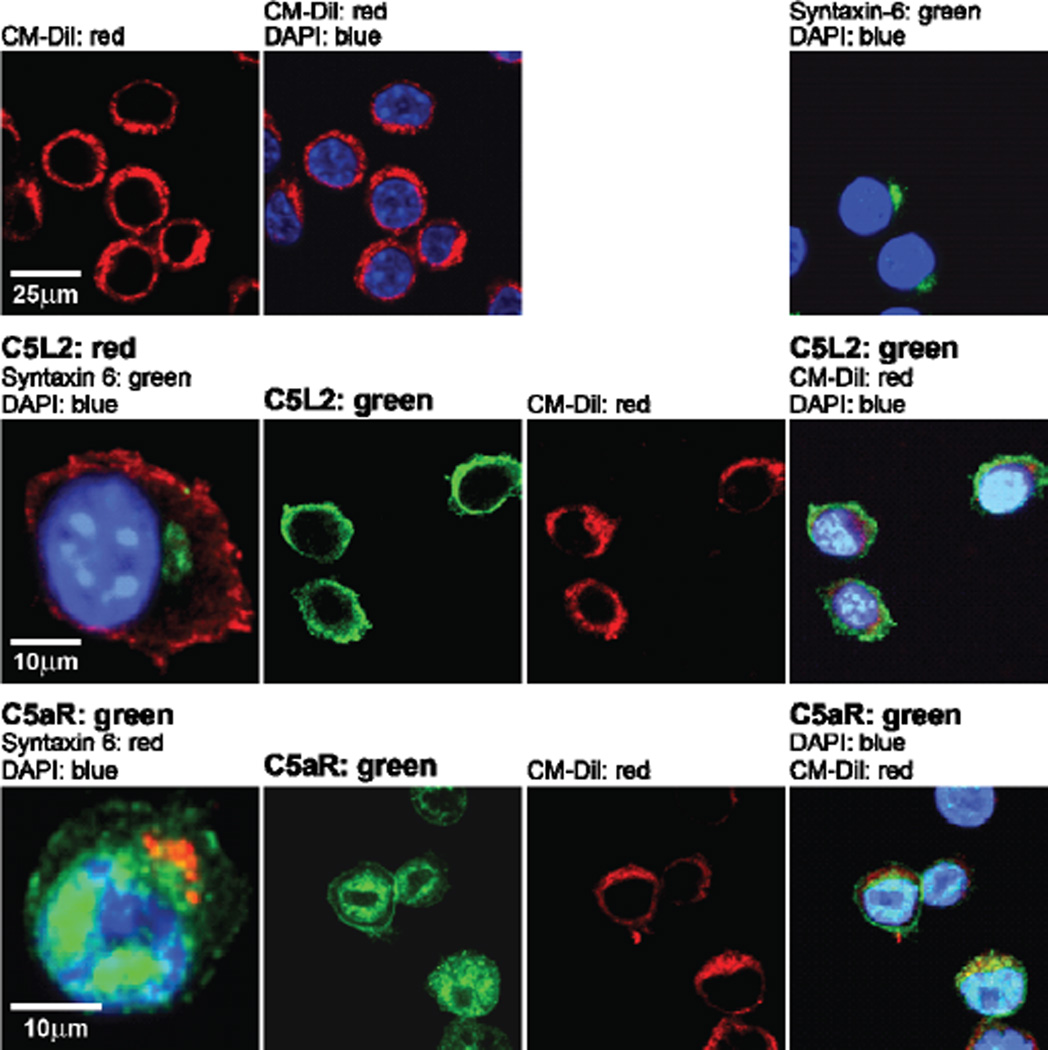
Subcellular distribution of C5L2 and C5a receptor (C5aR) in RAW 264.7 macrophages. C5L2 and C5aR were detected in RAW 264.7 cells by immunofluorescence using antibody against C5L2 or C5aR. Nuclei were stained with DAPI, syntaxin-6 was used as Golgi/ER marker and CM-Dil was used as cell membrane marker. Figures are representative of images from 3 independent experiments.

To understand the mechanism for MDP-mediated priming of macrophages to C5a, we first asked if MDP affected expression of the C5a receptors, C5aR or C5L2. Using confocal microscopy, the subcellular distribution of C5L2 and C5aR was characterized in RAW 264.7 macrophages. C5L2 was predominantly co-localized with CM-Dil, a cell surface marker (Figure 3), while C5aR distributed both to the cell surface and throughout the cytoplasm in a punctate distribution (Figure 3). No signal was observed in cells not incubated with primary antibody (data not shown). The subcellular distribution of C5L2 and C5aR in RAW 264.7 cells (Figure 3) was different from previous studies on human neutrophils where C5aR is located on the plasma membrane and C5L2 is predominantly expressed on intracellular vesicles [3].

**Figure 4:**
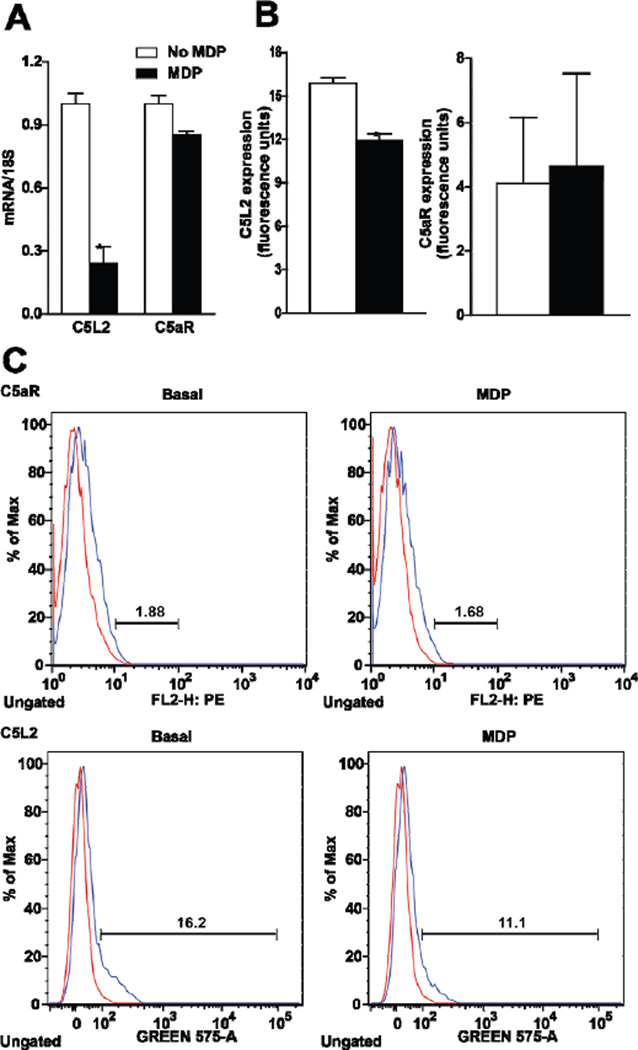
Differential regulation of C5a receptor (C5aR) and C5L2 in RAW 264.7 macrophages after long-term exposure to MDP. (A/B) RAW 264.7 macrophages were incubated with MDP (1 µg/mL) for 16 h. Expression of (A) C5L2 and C5aR mRNA, measured by qRT-PCR and (B) cell surface expression of C5L2 and C5aR protein, measured by flow cytometry. Values represent means ± SEM, *n* = 3 – 4, *p < 0.05 compared to cells not treated with MDP.

Since RAW 264.7 macrophages expressed both C5aR and C5L2, we next investigated whether priming with MDP affected expression of the C5a receptors, C5aR or C5L2. Sixteen hour treatment with MDP decreased expression of C5L2 mRNA in the cell, but had no effect on expression of C5aR mRNA (Figure 4A). MDP priming also suppressed C5L2, but not C5aR, quantity on the cell surface (Figure 4B). Since C5L2 acts as a negative modulator of C5aR activity [1], these data suggest that C5L2 plays a key role in synergism between C5a and NOD2 signaling.

**Figure 5:**
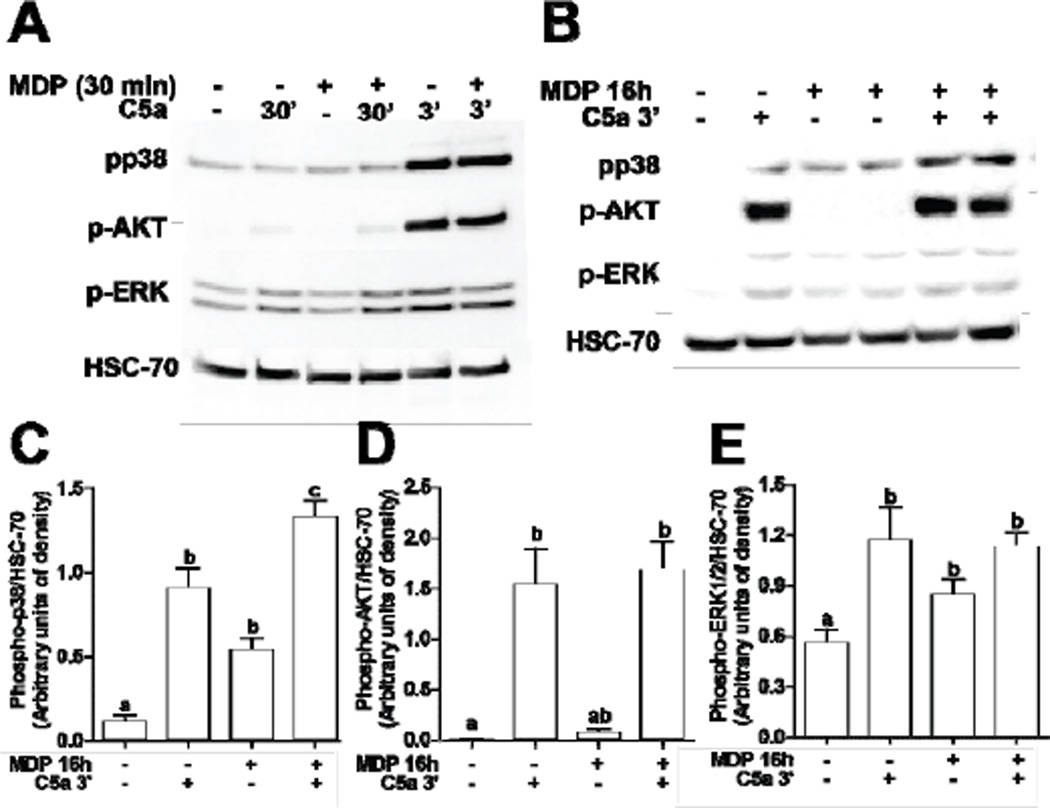
C5a-mediated signal transduction with and without concurrent or long-term exposure to MDP. RAW 264.7 macrophages were stimulated with MDP (1 µg/mL)for (A) 30 min or (B/C/D/E) 16 h and then challenged with C5a (10 ng/mL) for 3 or 30 min. Phosphorylation of AKT, p38, and ERK1/2 were measured by Western-blot analysis and normalized to HSC-70 as a loading control. (C/D/E) Values represent means ± SEM, *n* = 4 – 6. Values with different superscripts are significantly different from each other, *p* < 0.05.

In order to better understand the mechanisms by which NOD2 signaling enhanced C5a-mediated chemokine expression, C5a-mediated activation of kinases was assessed in RAW 264.7 macrophages. Challenge of RAW 264.7 macrophages C5a rapidly increased the phosphorylation of AKT, p38and ERK1/2 (Figure 5A), with phosphorylation evident by 3 min and declining by 30 min (Figure 5A). Short term exposure to MDP (30 min), prior to challenge with C5a, had no effect on C5a-stimulated phosphorylation of AKT or the MAP kinases (Figure 5A), consistent with the lack of synergism in cytokine/chemokine expression when both MDP and Ca were added to RAW264.7 macrophages simultneaously (Figure 1). In contrast, when RAW 264.7 macrophages were pre-treated with MDP for 16 h and then challenged with C5a for 3 min, the phosphorylation of p38 was increased compared to cells not pre-treated with MDP (Figures 5B, C).However, C5a-induced phosphorylation of AKT or ERK1/2 was not increased by priming with MDP (Figures 5B, D, E).

**Figure 6:**
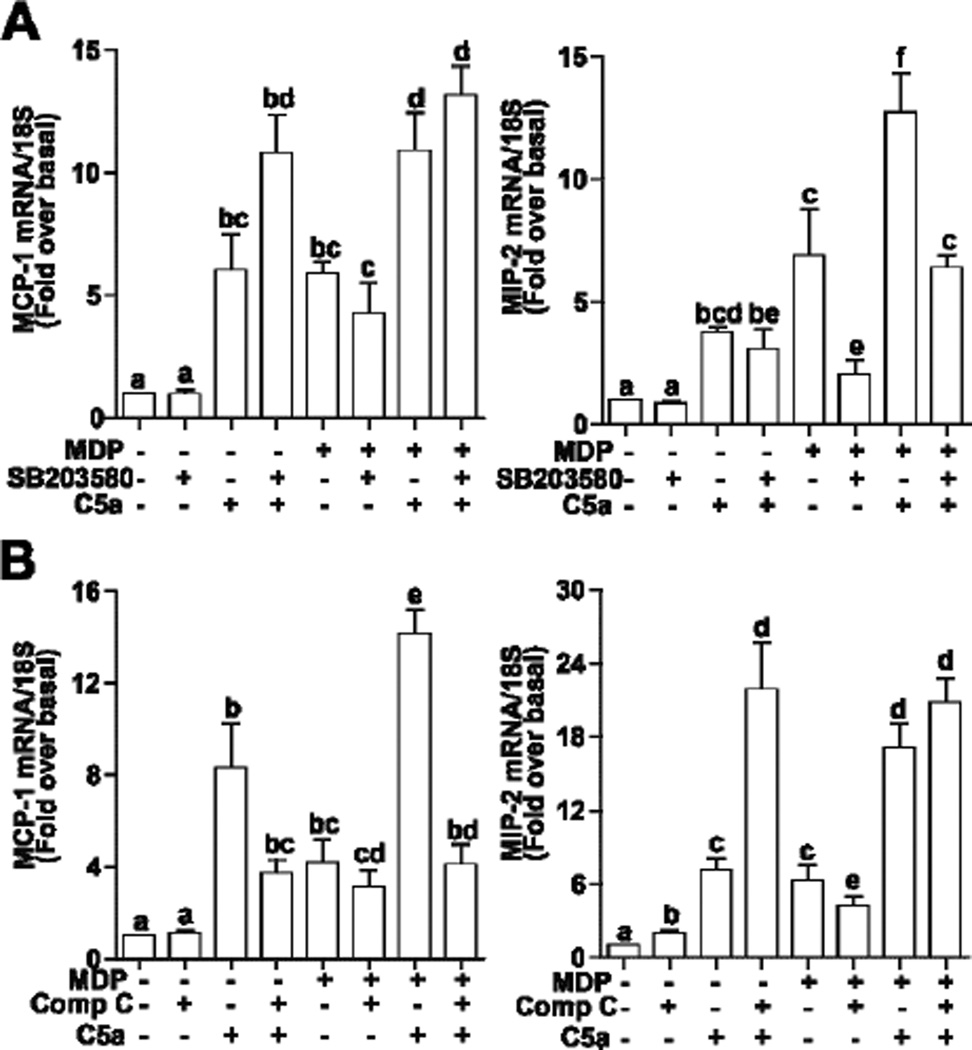
Differential contributions of AKT and p38 MAPK signaling to C5a-induced expression of chemokines in RAW 264.7 macrophages after long-term exposure to MDP. RAW 264.7 macrophages were incubated with MDP (1 µg/mL) 16 h. Cells were subsequently treated with or without (A) SB20358, an inhibitor of p38, or (B) compound C (Comp-C), an inhibitor of AKT, for 30 min and then challenged with or without C5a (10 ng/mL) for 90 min. Expression of MCP-1 and MIP-2 mRNA were measured by real-time PCR. Values represent means ± SEM, *n* = 4 – 6. Values with different superscripts are significantly different from each other, *p* < 0.05.

If these C5a-mediated signals were important for the regulation of chemokine expression after treatment with MDP, then inhibition of kinase activities should prevent enhanced C5a signaling in MDP-treated RAW 264.7 macrophages. RAW 264.7 macrophages were first pre-treated with MDP for 16 h and then media changed before cells were treated or not with SB20358, to inhibit p38 signaling, or compound C, to inhibit AKT signaling, for 30 min prior to challenge with C5a. This experimental design precluded any non-specific effects of SB20358 on NOD2 signaling [16] and instead focused the role of inhibitor only during the challenge with C5a. Interestingly, both SB20358 and compound C had differential effects on the synergistic interactions between MDP and C5a on MCP-1 and MIP-2 expression. Pre-treatment with SB20358 attenuated the sensitization of RAW264.7 macrophages to C5a after MDP exposure on MIP-2 mRNA expression, but had no effect on MCP-1 expression (Figure 6A). In contrast, compound C reduced sensitivity to C5a-induced MCP-1 production, but not MIP-2 (Figure 6B). Inhibition of ERK1/2 with PD98059 had no effect on the sensitization of C5a-mediated expression of MCP-1 and MIP-2 (data not shown). These data suggest that MDP sensitizes C5a-induced chemokine production via different mechanisms.

## 4. DISCUSSION

**Figure 7:**
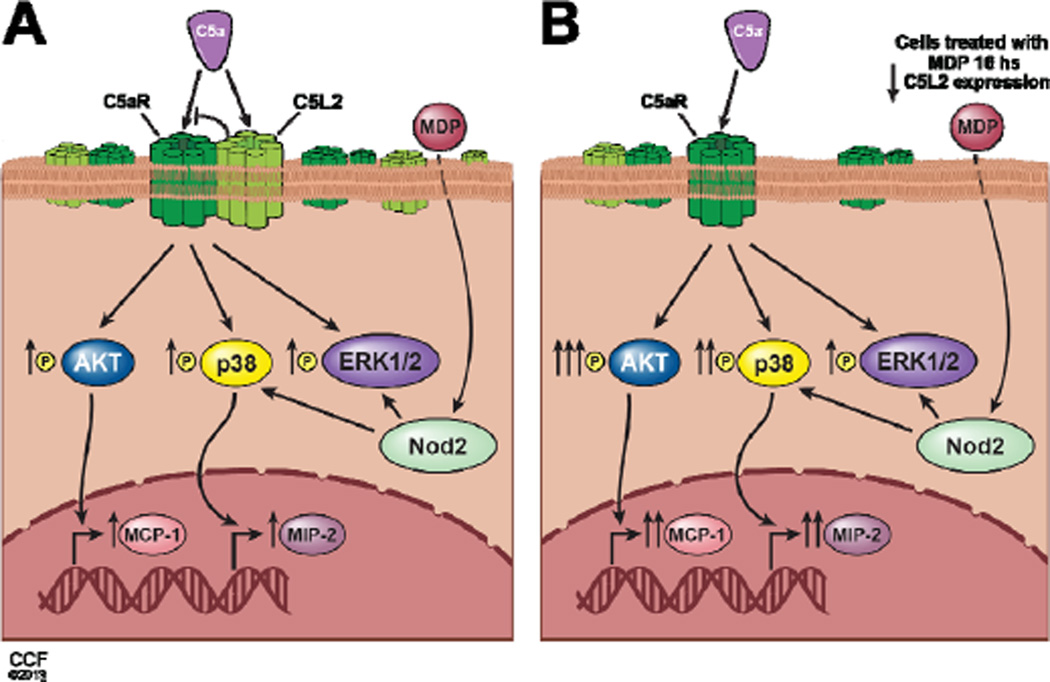
Synergistic interaction between C5aR signaling and NOD2 receptor signaling in macrophages. (A) C5a and MDP each stimulate chemokine expression in RAW 264.7 macrophages. (B) Priming with MDP, a NOD2 agonist, enhanced C5a- mediated response, associated with a decrease in the expression of C5L2, a negative modulator of C5aR activity.

Mounting of an innate immune response requires a coordinated activation and inactivation of multiple receptor-mediated signaling responses. While much attention has been paid to the interaction of TLRs with other innate immune pathways, the networking of complement anaphylatoxin signaling with other pathways has not been well studied. Complement signaling via C5aR can both activate and inhibit TLR4 signaling leading to the expression of inflammatory cytokines; this response is dependent on the cellular/physiological context [5,17,18]. Here we report for the first time a synergistic interaction between C5aR signaling and NOD2 receptor signaling in macrophages (Figure 7). When macrophages were primed with MDP, a NOD2 agonist, subsequent responses to C5a receptor activation were enhanced. Here we focused on the senstivity of RAW 264.7 macrophages to C5a-stimulated chemokine expression, as C5a itself is a powerful chemokine [19]. NOD2-dependent regulation of C5a signaling was associated with a decrease in the expression of C5L2, a negative modulator of C5aR activity [1], causing an increase in the ratio of C5a receptor to C5L2. These data add to the growing body of evidence for an important role for C5L2 in mediating the complex cellular responses to challenge with C5a.

C5a interacts with two receptors, the C5a receptor and C5L2. C5a binds both receptors with a similar affinity, while C5L2 binds to the C5a decay product, *des*-Arg-C5a with a higher affinity [20]. The C5a receptor is a classic G-protein coupled receptor and activates its signaling via heterotrimeric G proteins. In contrast, C5L2 receptor has homology to the G-protein receptor family, but studies have revealed that it is unable to interact with intracellular G proteins [20,21]. Thus, it is still unclear whether C5L2 couples to non-G-protein-dependent signaling mechanisms or acts as a ligand scavenger [2,22]. What is clear from the literature is that decreased C5L2 enhances pro-inflammatory signaling via C5a receptor. For example, C5L2 antibody blockade increases C5a-mediated signaling and chemotaxis in human neutrophils [3]. Evidence also indicates that regulation of C5L2 expression is an important mechanism for cross-talk between TLR and complement signaling; TLR4 activation leads to a down-regulation of C5L2 expression in PBMCs and enhances C5a-stimulated cytokine expression [1]. The present study reports that priming of macrophages with MDP to activate NOD2-dependent signaling also suppresses C5L2 expression and exacerbates macrophage responses to subsequent challenge with C5a.

In order for C5a receptor and C5L2 to interact and modulate C5a signaling, it would be predicted that both receptor should reside in similar subcellular compartments, either prior to binding of ligand and/or as a consequence of ligand binding. In human neutrophils, C5L2 is primarily localized to intracellular vesicles while C5a receptor is on the plasma membrane [3]. In response to C5a, C5a receptor is internalized and co-localizes with C5L2 [3]. In contrast, in RAW 264.7 macrophages, C5L2 was primarily localized to the cell surface, with C5a receptor localized to both the cell surface and intracellular vesicles (Figure 3). Since C5L2 was localized primarily at the cell surface in RAW 264.7 macrophages, it is likely that C5L2 may function as a ligand scavenger and prevent the interaction of C5a with C5a receptor at the cell surface of RAW264.7 macrophages.

C5a receptor signals via multiple pathways; MAPK and Akt-dependent signaling is particularly important in multiple cell types [23,24]. Challenge of RAW 264.7 macrophages with C5a rapidly stimulated phosphorylation of p38 and ERK1/2 kinases and also increased the phosphorylation of Akt. While treatment with MDP also increased p38 and ERK1/2 phosphorylation, with minimal direct effect on phosphorylation of Akt, there was a strong synergistic effect of MDP-priming on C5a stimulation on p38 MAPK, but not Akt. Making use of pharmacological inhibitors of these pathways only after the priming with MDP, our study design avoided non-specific interactions between the inhibitors and NOD2 signaling [16]. Using this design, we were able to distinguish the important contribution of both p38 and Akt to enhanced C5a-mediated chemokine expression. Interestingly, p38 and Akt contributed differentially to C5a- stimulated chemokine expression, with p38 critical to the expression of MIP-2, but not MCP-1, and Akt contributing to MCP-1 expression, but not MIP-2. Consistent with these data, previous studies also identified a critical role for p38 in C5a-dependent expression of MIP-2, dependent on the activation of C/EBPβ and C/EBPδ, in macrophages [25].

Taken together, the current data demonstrate an important interaction between NOD2 and C5a-mediated signaling. This interaction will likely be important to our understanding of chronic inflammatory diseases; the interaction between NOD2 and complement signaling may be of particular interest in Crohn’s disease. The role of the innate immune response in Crohn’s disease and inflammatory bowel disease is complex. While the disease itself is characterized by chronic inflammation, recent studies suggest that an early immunodeficiency may actually contribute to disease initiation and progression [26]. This apparent conundrum was first revealed upon the identification of the strong association of the CARD15 mutation in the IBD1 locus as a susceptibility locus for Crohn’s disease [8,9]. CARD15 encodes the cytoplasmic protein, NOD2. Surprisingly, this mutation was associated with a lowered response of NOD2 to activation by MDP [27]. Mutations in gene encoded Nod 2 account for 10% – 15% patients with this disease [10]. Similarly, while C5a receptor−/− mice were protected from acute colitis, the absence of C5a receptor resulted in more severe intestinal pathology in chronic models of colitis [28]. In light of this divergent role of innate immune activity in acute vs. chronic inflammatory bowel disease, our data suggest that impaired interaction between NOD2 and C5a receptor signaling may contribute to the onset of intestinal inflammation in Crohn’s disease patients, but after the onset of disease, may likely be an important therapeutic target for intervention to normalize chronic inflammatory responses.
